# Dialysis attendance patterns and health care utilisation of Aboriginal patients attending dialysis services in urban, rural and remote locations

**DOI:** 10.1186/s12913-022-07628-9

**Published:** 2022-02-24

**Authors:** Gillian Gorham, Kirsten Howard, Joan Cunningham, Paul Damian Lawton, A. M. Shamsir Ahmed, Federica Barzi, Alan Cass

**Affiliations:** 1 Menzies School of Health Research, Charles Darwin University, Darwin, 0810 Australia; 2 Sydney School of Public Health, Faculty of Medicine and Health, The University of Sydney, Sydney, Australia; 3 Menzies Centre for Health Policy and Economics, Faculty of Medicine and Health, The University of Sydney, Sydney, Australia; 4 Primary Health Tasmania (Tasmania PHN), Hobart, Tasmania Australia; 5 UQ Poche Centre, University of Queensland, Brisbane, Queensland Australia

## Abstract

**Background:**

Aboriginal people in the Northern Territory (NT) suffer the heaviest burden of kidney failure in Australia with most living in remote areas at time of dialysis commencement. As there are few dialysis services in remote areas, many Aboriginal people are required to relocate often permanently, to access treatment. Missing dialysis treatments is not uncommon amongst Aboriginal patients but the relationship between location of dialysis service and dialysis attendance (and subsequent hospital use) has not been explored to date.

**Aim:**

To examine the relationships between location of dialysis service, dialysis attendance patterns and downstream health service use (overnight hospital admissions, emergency department presentations) among Aboriginal patients in the NT.

**Methods:**

Using linked hospital and dialysis registry datasets we analysed health service activity for 896 Aboriginal maintenance dialysis patients in the NT between 2008 and 2014. Multivariate linear regression and negative binomial regression analyses explored the associations between dialysis location, dialysis attendance and health service use.

**Results:**

We found missing two or more dialysis treatments per month was more likely for Aboriginal people attending urban services and this was associated with a two-fold increase in the rate of hospital admissions and more than three-fold increase in ED presentations. However, we found higher dialysis attendance and lower health service utilisation for those receiving care in rural and remote settings. When adjusted for age, time on dialysis, region, comorbidities and residence pre-treatment, among Aboriginal people from remote areas, those dialysing in remote areas had lower rates of hospitalisations (IRR 0.56; *P* < 0.001) when compared to those who relocated and dialysed in urban areas.

**Conclusion:**

There is a clear relationship between the provision and uptake of dialysis services in urban, rural and remote areas in the NT and subsequent broader health service utilisation. Our study suggests that the low dialysis attendance associated with relocation and care in urban models for Aboriginal people can potentially be ameliorated by access to rural and remote models and this warrants a rethinking of service delivery policy. If providers are to deliver effective and equitable services, the full range of intended *and unintended* consequences of a dialysis location should be incorporated into planning decisions.

**Supplementary Information:**

The online version contains supplementary material available at 10.1186/s12913-022-07628-9.

## Introduction

The burden of chronic kidney disease disproportionately affects socioeconomically disadvantaged populations [1–3]. In Australia, the Northern Territory (NT) has the highest rates of kidney failure, with Aboriginal people who live in remote areas requiring kidney treatment at more than 10 times national rates [4].

The uptake of home therapies (self-care haemodialysis and peritoneal dialysis) is significantly lower in the NT compared to other Australian jurisdictions and few Aboriginal people receive a transplant. Thus most Aboriginal patients receive dialysis in a staffed facility [5]. Until recently there were few staffed dialysis services available in rural and remote areas [6] where the burden of kidney disease is heaviest. With renal and dialysis services centralised in the urban areas in the NT, most Aboriginal people with kidney failure must move, often permanently, for treatment.

Existing research indicates that a model of dialysis care that requires a person to permanently relocate away from their usual residence and support networks has significant negative consequences for the individual and their community [7–12]. Family and cultural responsibilities, coupled with distance and cost of remote travel often manifest in missed dialysis treatments in urban areas [13, 14]. Consistently lower rates of dialysis attendance are thought to be associated with higher hospitalisations [15, 16] but there has been little examination of the difference in dialysis attendance rates by location or how this may impact on other health service utilisation.

### Objective

We aimed to examine the relationship between the type and location of a dialysis model of care and broader health service utilisation, defined as dialysis treatments, hospital admissions, days in hospital and emergency department presentations. Of particular interest were patterns of health service utilisation for patients from remote areas who needed to relocate to receive care in urban locations, compared to patients who were able to access dialysis services closer to home.

## Methods

The NT is a large land mass of over 1.3million square kilometres with a small and sparsely dispersed population of fewer than 250,000 people. Most of the NT is classified as remote and very remote [17]. Most people live in the two main urban centres of Darwin and Alice Springs; but the majority (70%) of Aboriginal people, who make up approximately 30% of the population, live in remote and very remote communities.

**Fig. 1:**
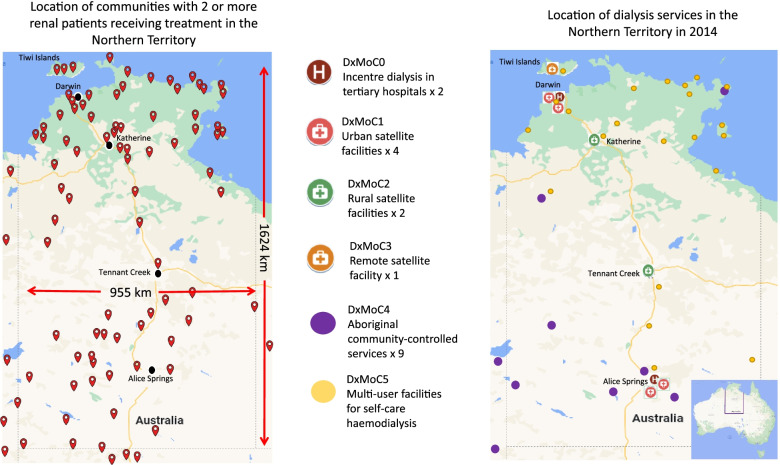
Residential location of kidney patients and location of NT dialysis services in 2014

Renal services are configured on a hub and spoke model with a hub based in the tertiary hospitals in Darwin and Alice Springs, 2000 km apart. The hubs oversee care delivered in urban, rural and remote satellite centres, notionally described as spokes. At the time of this study, most staffed dialysis services were centralised in the urban areas with only limited dialysis services available outside of these areas despite the significant demand. Renal patients relocate from more than 50 remote communities, including across jurisdictional boundaries, in order to access services (Fig. 1).

For the purposes of this study and ease of differentiation between services, we categorised dialysis services by location (urban, rural and remote) and support type and allocated services to a model of care (DxMoC). Table 1 describes the characteristics of the DxMoC depicted in Fig. 1. Transplantation is not a dialysis model of care and was not included in the study.

**Table 1:**
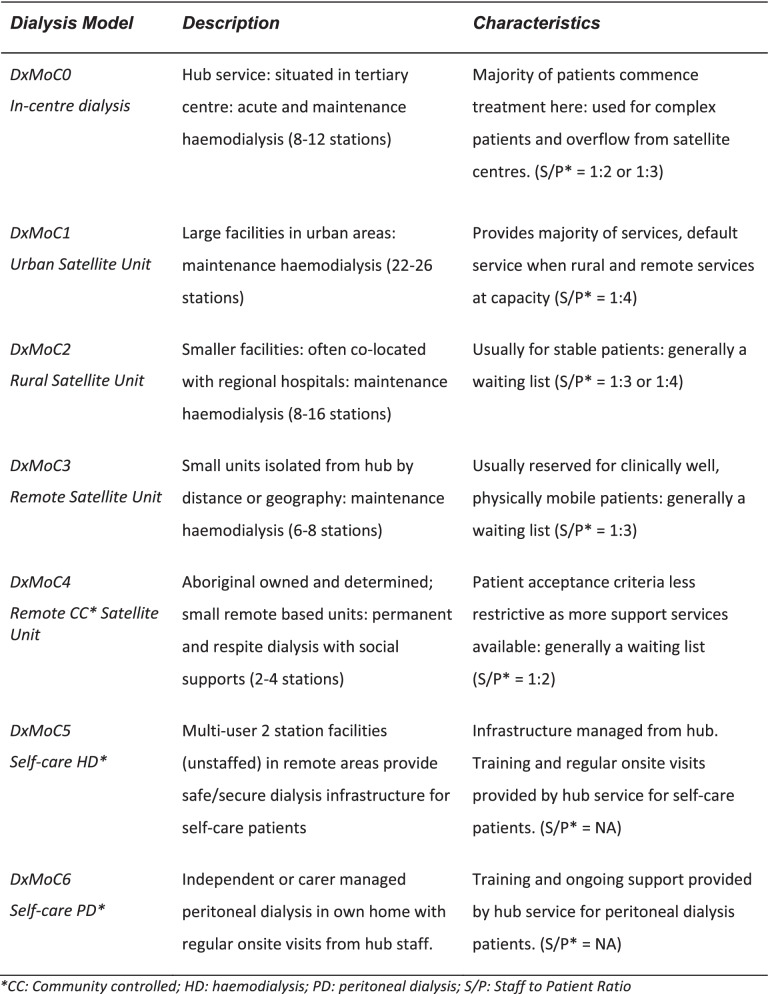
Dialysis services in the NT characterised as Dialysis Models of Care (DxMoC)

### Overall study design

We conducted a retrospective analysis of hospital and registry data to examine health service utilisation (dialysis attendance rates, hospital admissions, days in hospital and emergency department presentations) of maintenance dialysis patients in the NT between 2008 to 2014. The study received Ethics approval from the Joint Department of Health and Menzies School of Health Research Human Research Ethics Committee (HREC 2015–2334) and the Central Australian Health Research Ethics Committee (HREC 15–283).

### Data sources and linkage

We used two linked longitudinal data sets: the NT Department of Health’s (DoH) Admitted Patient Care (APC) (hospital) dataset which contains individual episodes of patient care for the five (public) hospitals and all satellite services in the NT; and the Australia and New Zealand Dialysis and Transplant Registry (ANZDATA) dataset which contains patient level data for people receiving maintenance kidney replacement therapy (KRT) in Australia and New Zealand. The ANZDATA dataset is based on a voluntary annual census from participating renal units.

The combined full database population (linked as part of the broader study [18]) included: 1) any individual from the APC dataset with a diagnosis or procedure code for dialysis or transplantation (ESM Table S1) based on the International Classification of Diseases version 10, Australian Modification (ICD 10 AM) between the years 2000 and 2015 (*n* = 2844); and 2) any individual from the ANZDATA dataset who registered as ever having KRT in the NT between 2000 and 2015 (*n* = 1390).

Linkage of two additional activity data sets was necessary: a) interstate patient travel information (*n* = 171); and b) dialysis data from individuals (*n* = 189) receiving self-care haemodialysis (DxMoC5) and/or care in the remote community-controlled service (DxMoC4), as this information was known to be inconsistently captured in the hospital data set.

### Study cohort definition

This study included Aboriginal adults (over the age of 16 years as of 1st Jan 2008), receiving maintenance dialysis (defined as chronic haemodialysis or peritoneal dialysis continuously for greater than 3 months), between 2008 and 2014 inclusive. This date range was chosen as some models of care only became fully established after 2008 and the additional activity data (for DxMoC4 and DxMoC5) was only available to the end of 2014.

**Fig. 2:**
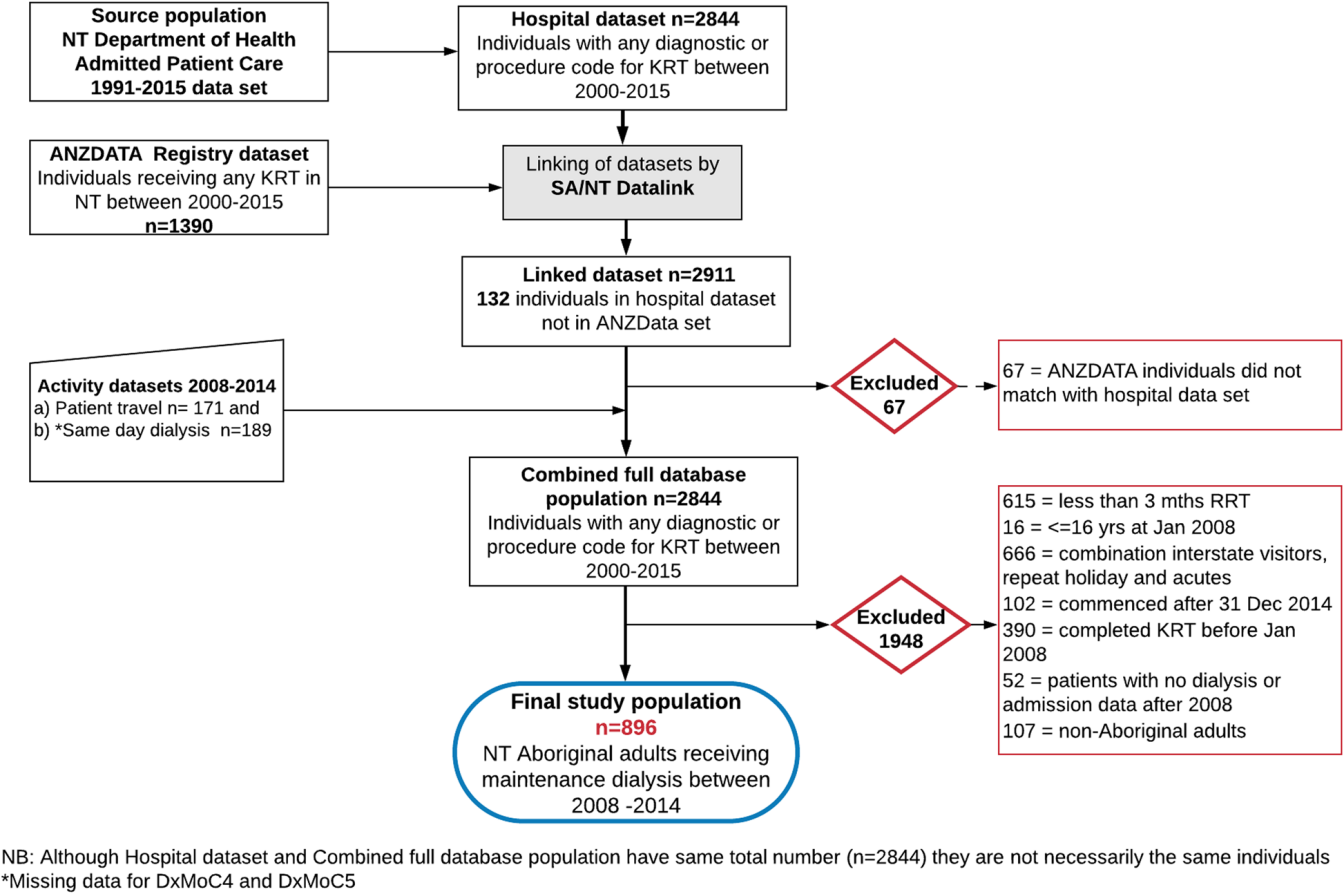
Patient selection flow chart 2008–2014

Non-Aboriginal dialysis patients were excluded as they comprised less than 10% of the dialysis population and were not represented in three (rural and remote models) of the seven models of care. Patients were also excluded if: they received acute dialysis treatments only; were interstate visitors; or did not have at least one admission after 2008, to exclude patients who left the NT permanently. Patients were censored if they had an active transplant (Fig. 2).

Additional information regarding setting, data sources, linkage and methods can be found in the Electronic Supplementary Material (ESM).

### Variable definitions

Besides the exposure variables of gender, age at time of admission, time on dialysis at admission, we were also interested in region (Top End or Central Australia to align with health service responsibility); residence pre-KRT (defined as the earliest admission address in the 24 months pre-KRT start); and relocation (defined as an indefinite change in residence from remote area pre-KRT to an urban address post commencement of maintenance dialysis). Our process may have underestimated ‘relocation’ as many remote residing people will move closer to specialist care as their health needs increase but before they require KRT [19]. Residences were mapped to the Modified Monash Model (MMM) which categorises areas according to remoteness [20]. See ESM for further details.

Selected comorbid conditions (diabetes, cardiac disease, hypertension, vascular disease, cerebrovascular disease and obesity) were flagged for each admission, based on the presence of relevant ICD 10 AM codes (ESM Table S2). Once present, a condition was carried forward to subsequent episodes.

Other exposure variables included the dialysis model of care (DxMoC) and dialysis attendance, which was both an exposure and outcome variable.

The dialysis model of care was determined through a combination of admission ward, treatment option based on ICD 10 AM diagnosis or procedure codes (ESM Table S3) and ANZDATA treatment coding to determine the ‘best fit’. As the intent of our study was to explore the relationship between dialysis model and subsequent health care use, determining the dominant model was critical and needed to accommodate the frequent movement of individuals between both facilities and modalities. This ws particularly important for the remote community-controlled model (DxMoC4) which operated primarily as a staffed reverse respite model, enabling individuals originally from remote areas to have short periods (2–6 weeks) of dialysis in their own community. Therefore, the dominant DxMoC was based on the model in which the majority of attendances had occurred in the current and preceding two-week period, rolling forward a week at a time. This process, while eliminating extremely frequent movements between models, still enabled the respite model (DxMoC4) of 2–6 weeks to be captured in the data.

Exposure to a model was then based on the time (in weeks) spent in the dominant DxMoC on an annual basis. Exposure time was censored at death or permanent loss to follow-up, defined as absence of any activity (dialysis or hospital admissions) without re-appearance before study end.

Dialysis attendance rates were calculated from the total attendances (based on 3 x week or a proportion thereof) while in a DxMoC and divided by the exposure time (in weeks) for that model. This was then multiplied by fifty-two (52 weeks) to calculate an attendance rate per year per DxMoC. The calculation excluded time as an inpatient, time as a transplant patient and time interstate, as identified from the patient travel data.

Dialysis attendance was also categorised using three levels:

- ‘High’ (greater than or equal to 144 treatments a year from 156 prescribed treatments, i.e. missing one or fewer/month)
- ‘Medium’ (between 132 and 143 treatments, i.e. missing between one and two/month) and
- ‘Low’ (less than or equal to 131 treatments a year, i.e. missing more than two/month).

Definition of outcome and exposure variables are available at ESM Table S4.

Hospital admissions, Emergency Department (ED) presentations and days in hospital were summed by year and by model to provide respective utilisation estimates per person per model of care. We did not differentiate hospital admissions and ED presentations that occurred in the urban tertiary hospitals from the rural hospitals. We also examined diagnosis codes for each admission episode and isolated those likely to be associated with low dialysis attendance (based on ICD-10 AM codes for Fluid Overload (E87.7) and Hyperkalaemia (E87.5)). These were summed by year and model.

A small number of incorrectly linked episodes (*n* = 5 patients) were excluded. Nine (*n* = 9) haemodialysis patients had a gap of 12 months or more in the dataset with no haemodialysis attendance or hospital admission episodes, followed by a subsequent attendance. This gap was categorized as *intermittent* loss to follow up to cater for people who had extended periods interstate for medical care or were returning to dialysis after a failed transplant. Intermittent loss to follow up periods were not included in the model exposure calculation. Duplicate attendances (primarily from the overlap of dialysis attendance in the hospital data set and the additional, manually compiled data set for DxMoC4/5) were removed.

### Statistical analysis

All analyses were conducted using Stata15.1© (StataCorp., College Station; Texas USA). Simple linear regression analyses examined the relationship between annual dialysis attendance and gender, relocation, region, remoteness of residence pre-KRT start, age at admission, time on dialysis and DxMoC. Using both forward variable selection and backward variable elimination multivariate linear regression analyses, we tested the variables’ tolerance for inclusion or exclusion in the model based on the R squared and confidence intervals. Sensitivity analysis included a range of interaction terms between DxMoC and other variables such as remoteness pre-KRT start, relocation, region, time on dialysis, admission age as well as select comorbidities.

Negative binomial regression was used to model the outcome variables of hospital admission rates and ED presentation rates and corresponding 95% confidence intervals (CI) with all exposure variables mentioned above, as well as dialysis attendance as an exposure variable. Zero inflated poisson regression was used to model days in hospital, due to the frequency of zero counts.

## Results

### Patient characteristics

The final study population consisted of *n* = 896 Aboriginal maintenance dialysis patients between the years 2008 to 2014. The cohort consisted of more females than males and most of the study population (82%) were flagged as having relocated to start treatment. The age at start of KRT and median study observation time were similar for men and women (Table 2).

**Table 2:**
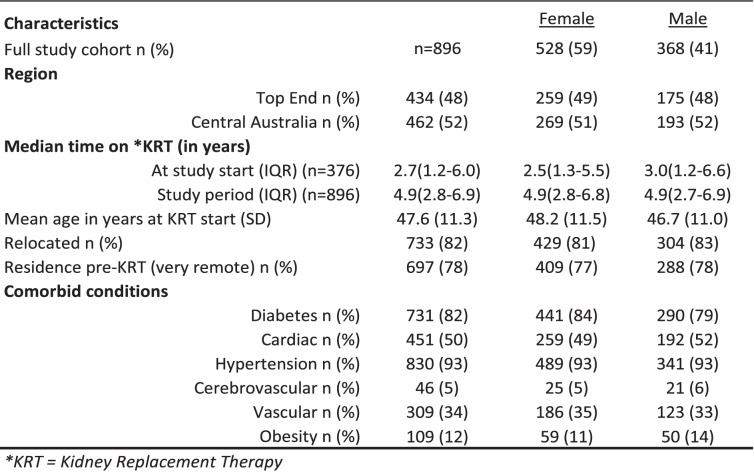
Patient characteristics at study entry of NT Aboriginal KRT patients, 2008–2014

To assess potential differences in patient mix by DxMoC, we compared mean age and presence of comorbidities for each DxMoC. There was little difference across models for either age or comorbidities, although individuals attending In-centre models (DxMoC0) were generally younger than those attending other models. The proportion of individuals with diabetes and vascular disease were also lower at In-centre DxMoC0 compared to the other models (Table 3). Full comorbidities by model of care are shown in ESM Table S5.

**Table 3:**
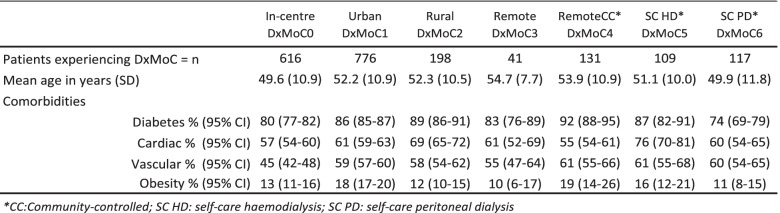
Characteristics of Aboriginal patients at time of admission to DxMoC, 2008–2014

### DxMoC and Dialysis attendance

**Fig. 3:**
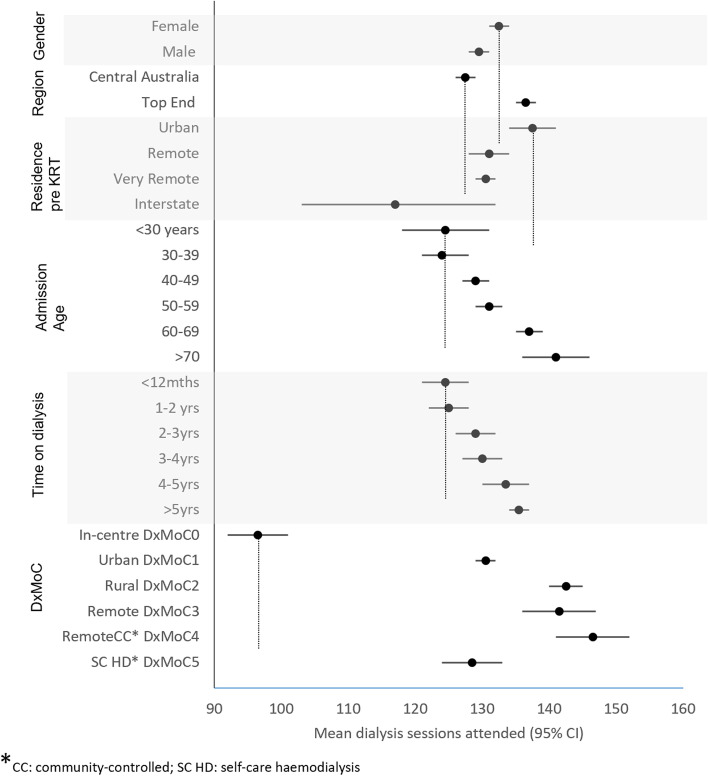
Mean annual dialysis attendances for NT Aboriginal patients, 2008–2014 – adjusted for all variables shown

Gender, remote residence pre-KRT start, patient age (less than 59 years), living in Central Australia (CA) and time on dialysis of less than four years were individually associated with lower yearly dialysis attendance (< 132 treatments) as was dialysing at the In-centre model compared to all other models (mean 129-147 vs 97, P<0.001). Full results are presented in ESM Table S6. When adjusted for gender, region, residence pre-KRT, admission age, time on dialysis and DxMoC, these associations persisted (Fig. 3).

### Dialysis attendance and hospital admissions

When dialysis attendance was stratified into a categorical variable, we found 43% of all attendances fell into the ‘High’ (> = 144 treatments), 15% into the ‘Medium’ (132–143) and 42% into the ‘Low’ (<=131 treatments) attendance categories although there was substantial variation by DxMoC. The proportion of attendances that fell into the ‘High’ category were highest in DxMoC2,3 and 4 while In-centre DxMoC0 had the highest proportion of ‘Low’ attendances followed by Urban DxMoC1.

On examination of the reasons for hospitalisations, (based on Australian Refined Diagnosis Related Groups [21]), we noted that while the majority (95%) of admissions contained a renal related diagnosis code, 25% of all admissions included one or both ICD-10 AM code for fluid overload (E87.7) or hyperkalaemia (E87.5) and a very high proportion of patients (78% or *n* = 695) had at least one admission with the above code. While admissions for fluid overload/hyperkalaemia can be unrelated to missing haemodialysis treatments, these coded admissions were higher for patients who had ‘Low’ dialysis attendance (72%) compared to ‘High’ dialysis attendance (13%). The proportion of admissions by DxMoC containing these codes (which we categorised as ‘Missed Dialysis’) were highest in the urban models (Table 4). Mean (with 95% CI) and median (with range) hospital admissions by an individual’s exposure time to a specific DxMoC are also presented in Table 4.

**Table 4:**
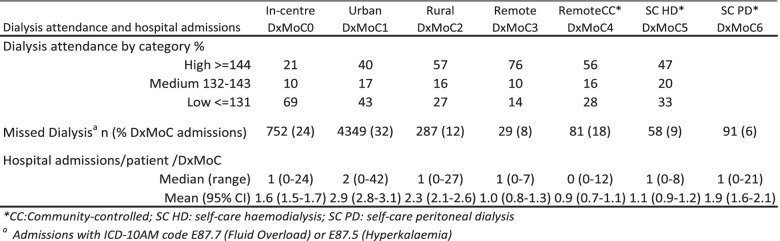
Dialysis attendance and hospital admissions for NT Aboriginal patients by DxMoC, 2008–2014

Multivariate negative binomial regression showed a significant increased rate of both hospitalisations (IRR=2.10; 95% CI:1.96-2.28) and ED presentations (IRR=3.29; 95% CI :2.86-3.80) associated with ‘Low’ dialysis attendance (<132 treatments) compared to high dialysis attendance (>143 treatments). Results are presented in ESM Table S7.

### DxMoC, hospital admissions and ED presentations

We modelled overnight hospital admissions and ED presentations separately, as an outcome with a range of exposure variables (without dialysis attendance and with self-care PD DxMoC6) using univariate and multivariate negative binomial regression. Unadjusted, region, remoteness of residence pre-KRT start, age less than 40 years, time on dialysis of less than four years, comorbidities of diabetes, cardiac disease, vascular disease and obesity as well as dialysing at In-centre DxMoC0 were all associated with an increased rate of overnight hospital admissions. These associations persisted in the multivariate analysis except for obesity, which was no longer significant, while time on dialysis greater than 12 months, was now associated with reduced rates of hospitalisation. We used Urban DxMoC1 as the reference due to the higher patient numbers attending this model (Table 5).

**Table 5:**
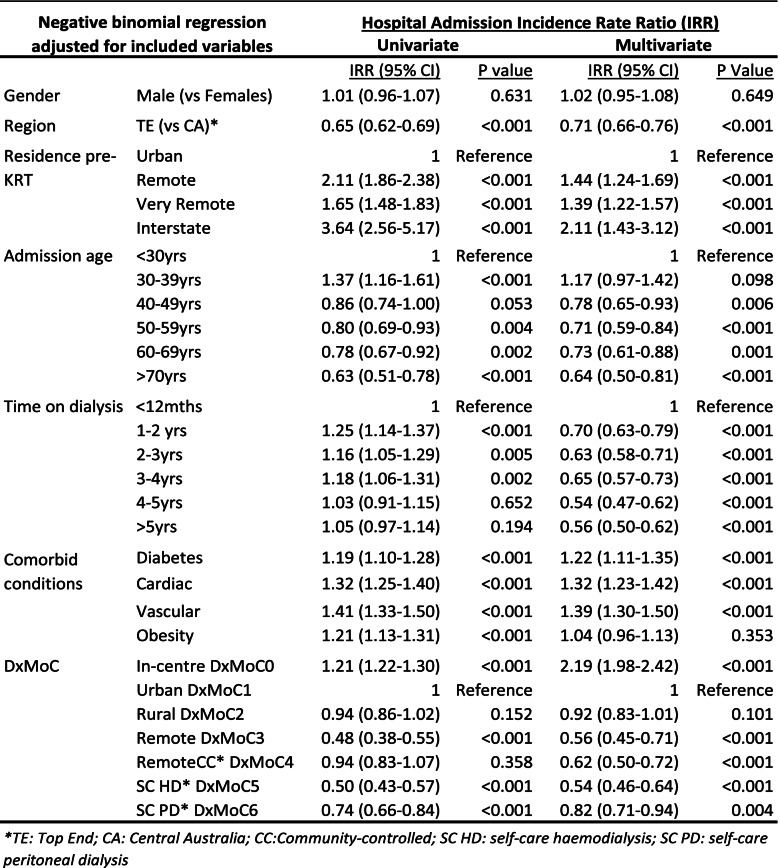
Hospital admission incident rate ratios (IRR) for NT Aboriginal patients, 2008–2014

Results for ED presentations are presented in Supplementary materials (ESM Table S8) and found generally similar results with respect to significantly lower rates of ED presentations for rural and remote dialysis models of care. Results for days in hospital are also presented in Supplementary materials (ESM Table S9).

## Discussion

Renal patients have high all-cause mortality and hospitalisation rates [22], usually related to cardiovascular events and infection. However we also showed a relationship between dialysis attendance, hospitalisations and DxMoC.

Our analysis of linked hospital clinical and administrative data for Aboriginal dialysis patients found a clear relationship between the location/type of treatment and dialysis attendance, and in turn these were both strongly associated with health service utilisation, particularly overnight hospital admissions. Our analysis did not include non-Aboriginal people as very few in the NT are from remote areas and the impact of relocation on health service utilisation and outcomes is not relevant to their experience of dialysis.

The relationship between missed or shortened dialysis treatments and hospitalisations has been explored previously. In one US study, missed and shortened treatments for a very small proportion of the study population (2.4%) were associated with progressively more hospitalisations although the relative risks (RR) were not provided [16]. A comparable Australian study described a significant and consistent increase in hospitalisations associated with missing one treatment a week (i.e. four per month) with an IRR = 1.90 in the first year [23]. Our analysis found that missing two or more treatments per month was associated with a two-fold increase in the rate of hospital admission and more than three-fold increase in the rate of ED presentations. Additionally, the proportion of patients who missed one or more dialysis attendance a month was relatively high at 42% or greater, compared to the United States (US) where 7.9% (in the Dialysis Outcomes and Practice Patterns Study) was considered high [16].

The relationship between the location of dialysis treatment, missed dialysis treatments and hospitalisation rates has not been extensively explored to date. Our study included an analysis of geographical factors relating to original residence and location of service. We found relocation and remoteness of residence pre-KRT start, as well as younger age, were associated with lower dialysis attendance, a finding that is not uncommon in Northern Australia [13].

‘Region’ was also a strong predictor of health service utilisation, with Central Australian patients 30% more likely to be admitted overnight and with a 70% higher risk of an ED presentation compared to patients in the Top End. These differences persisted after adjusting for age, time on dialysis, comorbidities and DxMoC.

However we found higher dialysis attendance and lower hospital admissions and ED presentations for Aboriginal patients receiving care in rural, remote and self-care haemodialysis models. While rural and remote services are usually reserved for clinically stable patients, we found no significant difference in the characteristics (age or comorbidities) of patients attending urban versus rural and remote models of care, indicating that age and the presence of comorbid conditions do not completely account for the higher rates of hospital admissions seen at In-centre DxMoC0 and Urban DxMoC1. Perhaps it also suggests that the low dialysis attendance associated with relocation and care in urban models can potentially be ameliorated by access to rural and remote models.

Patients being treated with self-care haemodialysis (DxMoC5) had lower dialysis attendance patterns on average but not increased health service utilisation. Although we made additional efforts to capture all relevant data, we believe the lower dialysis attendance for this group may have resulted from missing data.

To date, evidence on how models of care influence the quality of care and patient outcomes has been variable. Some evidence suggests that hospital presentations, along with an increased risk of morbidity and mortality, are associated with specific models of care, particularly remote based facilities where there is low access to nephrologists and significant distance to a tertiary service [24, 25]. However, in many regions/jurisdictions, movement between satellite units and hospitals is common, with an increased risk of hospitalisation associated with certain satellite facilities, not necessarily remote facilities [26] and not necessarily related to missed treatments [27].

Given the vastness of the NT, the number of remote communities and the limited services in rural and remote areas, there are bound to be differing degrees of relocation. People receiving services in rural areas may still have relocated from a very remote area. They may therefore face the same issues relating to the cost and time of travel to attend community activities, leading to missed treatments. The examination of attendance patterns and health service use by the degree of dislocation is an area for future analysis.

Our study found reduced rates of health service utilisation associated with dialysing in rural and remote locations. There is an argument that for remote living individuals, treatment within community improves outcomes [7, 28]. Patients maintain that these models, which provide care closer to home, are more supportive of the health and wellbeing of individuals, which in turn facilitates more active engagement with treatment [12, 29–32] leading to lower health service utilisation.

### Limitations

We are aware that validation studies for the use of ICD-10 coding to identify kidney disease (usually acute kidney injury or chronic kidney disease) have indicated poor congruence between diagnosable conditions and documentation [33, 34] and acknowledge that the coding of peritoneal dialysis (PD) and transplantation is likely to be of similarly reduced quality. The linkage with the ANZDATA set, however, has addressed this limitation. The coding of haemodialysis in the NT is relatively robust as it is the primary reason for admission and required for activity-based funding. Additionally, recent local validation studies in the NT improved the quality of this coding [35]. We also took extra steps to assess the validity of the data and source additional data sets where there was prior knowledge of data gaps. However we did not interpolate data where data gaps remained, as we could not be certain activity had occurred. In retaining the participant, such data gaps were designated *intermittent LTFU*.

Although we found few differences in age or presence of comorbidities across the models, we recognise that clinician and patient self-selection may limit access to remote models for more frail patients, particularly where there is limited facility capacity and waiting lists exist. We also recognise that low dialysis attendance is but one cause of poorer health and higher hospitalisations; renal patients are known to experience high rates of hospitalisations due to other factors, usually arising from complications associated with cardiac disease and sepsis [22, 36]. Nevertheless, our results suggest a statistically and clinically significant impact of dialysis attendance on hospitalisations and ED presentations.

## Conclusion

There is a significant gap in knowledge concerning the relationship between the provision and uptake of dialysis services in urban, rural and remote areas and subsequent broader health service utilisation. In the NT, Aboriginal people from remote areas have a very heavy burden of kidney failure requiring kidney replacement therapy.

The situation necessitates a re-examination of service provision and the identification of ways to deliver accessible, equitable and high-quality services that meet patients’ health, social and cultural needs.

This study highlights the differences in the attendance patterns and health service use associated with dialysis treatment in different locations and particularly for Aboriginal people originally from rural and remote areas. More broadly, this analysis also illustrates the impact of policy decisions on health service utilisation. Decisions to limit the variety of models of dialysis care available to patients may be understandable, particularly in remote areas, given constraints related to volume/demand, infrastructure capacity and costs of service provision. However, our analysis suggests that policy decisions that do not consider and appreciate the full range of intended *and unintended* consequences, including downstream health service utilisation, of various service models, may lead to suboptimal decisions about allocation of scarce resources.

## Supplementary Information

**Additional file 1.**

## Data Availability

The data that supports the findings of this study were provided by the Northern Territory Department of Health, but restrictions apply to the availability of these data, which were conditions of the Ethics approval and so are not publicly available. With permission of the Northern Territory Department of Health, data are however available from the authors upon reasonable request.
