# Supplementary material for: Dialysis attendance patterns and health care utilisation of Aboriginal patients attending dialysis services in urban, rural and remote locations
Source: BMC Health Serv Res. 2022 Feb 24;22:251. doi: 10.1186/s12913-022-07628-9 (PMC8867655; doi:10.1186/s12913-022-07628-9)
Supplement: Supplementary file 1 — Additional file 1. [file 12913_2022_7628_MOESM1_ESM.pdf]

Title:

Electronic Supplementary material for manuscript *'Dialysis attendance patterns and health care utilisation of Aboriginal patients attending dialysis services in urban, rural and remote locations'*

Journal:

Submission to BMC Health Services Research

Authors:

Gillian Gorham[1], Kirsten Howard[2,3], Joan Cunningham[1], Paul Damian Lawton[1], AM Shamsir Ahmed[4], Federica Barzi[5], Alan Cass[1].

[1]Menzies School of Health Research, Charles Darwin University, Darwin Australia.

[2]Sydney School of Public Health, Faculty of Medicine and Health, The University of Sydney, Sydney Australia.

[3]Menzies Centre for Health Policy and Economics, Faculty of Medicine and Health, University of Sydney, Sydney, Australia

[4]Primary Health Tasmania (Tasmania PHN) Hobart Tasmania, Australia

[5]UQ Poche Centre, University of Queensland, Brisbane Queensland, Australia

Corresponding author:

Gillian Gorham

Menzies School of Heath Research; PO Box 41096; Casuarina 0810; Darwin Australia

ORCID ID: 0000-0002-9814-2204
Email: gillian.gorham@menzies.edu.au;

Phone: +61 8 8946 8529

Electronic Supplementary Material Methods and Results

This supplement includes additional information relating to the methods and results for the dialysis attendance and health care utilisation analysis including cohort definition and data management.

Setting

Most of the Northern Territory in Australia is classified as remote and very remote according to the Australian Statistical Geography Standard (1) which classifies areas according to degrees of remoteness. Only Darwin is identified as Outer Regional. For the purposes of this study and ease of differentiation between services, we classified treatment locations into three categories (urban, rural and remote) based on access to health services and the Australian Governments classification of hospitals (2):

- Urban: Darwin and Alice Springs serviced by Principal Referral or Acute Group A Hospitals.
- Rural: Katherine and Tennant Creek serviced by small Acute Group C Hospitals
- Remote: all other treatment centres without hospitals.

Methods

Australia's health system is a mixture of publicly and privately funded services. Dialysis treatments in the Northern Territory are fully publicly funded under a case mix model based on coded discharge hospital data. All haemodialysis treatments, including those conducted in satellite facilities, are entered into the NT Department of Health's (DoH) Admitted Patient Care (APC) (hospital) dataset. There is only one private hospital in the NT, which does not provide dialysis treatments. Therefore the capture of dialysis activity in the NT is considered to be comprehensive and robust.

The full database population for this study was derived from the DoH's APC (hospital) dataset combined with the Australia and New Zealand Dialysis Transplant Registry (ANZDATA) dataset.

The APC hospital dataset contains individual episodes of patient care for the five (public) parent hospitals and several satellite services in the NT, from the beginning of consistent electronic record keeping (1991). It includes demographic details of the individual (age, ethnicity, residence) and the hospital (hospital code, ward/s), as well as admission/separation codes and diagnosis and procedure codes (primary and up to 49 secondary codes) based on the International Classification of Diseases version 10, Australian Modification (ICD 10AM). ANZDATA is the data repository for people receiving maintenance kidney replacement therapy (KRT) in Australia and New Zealand and contains patient level administrative and clinical data, based on an annual census from participating renal units. All renal units in the NT participate in the census.

The full database population included: 1) any individual from the APC dataset with an ICD 10AM diagnosis or procedure code for dialysis or transplantation (Table S1) between the years 2000 and 2015 (n= 2844); and 2) any individual from the ANZDATA dataset who registered as ever having dialysis in the NT between 2000 and 2015 (n=1390).

Table S1 includes the ICD 10AM diagnostic and procedure codes used to identify KRT admissions and create the 'database population' from the NT Department of Health Admitted Patient Care Hospital Dataset 1991-2015.

Table S1: ICD 10AM codes used to identify database population from hospital dataset

**ICD 10AM Procedure Code**
13100-00=Haemodialysis
13100-01=Intermittent Haemofiltration
13100-02=Continuous Haemofiltration
13100-03=Intermittent Haemodialfiltration
13100-04=Continuous Haemodialfiltration
13100-06=Peritoneal Dialysis (PD) short term
13100-07=Intermittent PD long term
13100-08=Continuous PD long term
13109-00=Insertion and fixation of PD catheter
13109-01=Replacement of indwelling catheter
13110-00=Removal of indwelling catheter
13112-00=PD with temporary catheter
36503-00=Kidney transplant
36503-01=Reimplantation of kidney
90351-00=Removal of temporary PD catheter
**ICD 10AM Diagnosis Code**
T85.71=Infection and inflammation reaction due to PD catheter
T86.1=Transplant rejection
Y84.1=Kidney dialysis (Other medical procedure as a cause)
Z49.0=Preparatory care for dialysis
Z49.1=Extracorporeal dialysis
Z49.2=Other dialysis
Z94.0=Kidney transplant status
Z99.2=Dependence on renal dialysis

The two datasets were linked by a third-party jurisdictional data linkage agency (SA/NT Datalink) following standard ethical systems and protocols. SA/NT Datalink is an independent agency based at the University of South Australia. Using probabilistic matching, de-identified individuals across data sets were linked and assigned a unique identifier. Due to the voluntary nature of the ANZDATA collection, a one to one (1:1) match with the hospital dataset was not expected and one hundred and thirty-two (132) individuals in the hospital dataset were not present in the ANZDATA set. Sixty-seven (67) individuals in ANZDATA dataset did not match any individuals in the hospital data set. These 67 individuals were excluded as hospital activity data was not available for analysis. The datasets included all hospital admission and registry data for eligible patients.

The full database population of 2844 individuals was then linked with activity data from two additional data sets: a) interstate patient travel information (n=171 patients); and b) dialysis data from individuals (n=189) receiving care in the community controlled DxMoC4 and self-care HD DxMoC5. This was necessary because inconsistencies in data entry for these models led to some gaps in attendance data, however, manual compilation of activity between 2008-2014 was possible and linkage with the hospital data set was undertaken by an independent linker not associated with the project.

Study cohort definition

The final study population (n=896) included individuals who had any KRT for more than three months continuously (to eliminate acute and short term dialysis support including patients visiting from elsewhere on holidays), between the years 2008 to 2014. This date range was chosen as some models of care only became fully established after 2008 and the additional activity data (for DxMoC4 and DxMoC5) was provided to the end of 2014. Restricting the analysis of patterns of health service utilisation to 2008 to 2014 ensured that sufficiently robust activity data across all models was available for the analysis.

Patients were excluded if they were 16 years and younger at 2008 or did not have at least one admission after 2008 – to exclude patients who left the NT and were therefore not eligible for inclusion. Non-Aboriginal patients (n=107) were also excluded as they comprised less than 10% of the population and did not experience all models of care. All available admission data pre 2008 was also retained for the purposes of identifying home residence and health status (comorbidities) prior to commencing dialysis.

Table S2 identifies the ICD 10AM codes (collapsed to group level) used to identify and map comorbid conditions for each admission. Once a comorbid condition was present it was carried forward to other admissions if not already present. Comorbidities were chosen for their relevance as kidney disease risk factors and concurrent chronic diseases considered to have a significant impact on health outcomes of kidney patients.

Table S2: ICD 10AM codes used to identify presence of selected comorbid conditions for each admission in Final Study Population 2008-2014

Variable	ICD 10 Group	High level description
**Diabetes**	E10-E14	Diabetes mellitus
	O24	Diabetes mellitus in pregnancy
**Obesity**	E65-E68	Overweight, obesity and other hyperalimentation
**Hypertension**	I10-I15	Hypertensive diseases
**Cardiac disease**	I20-I25	Ischemic heart diseases
	I26-I28	Pulmonary heart disease and diseases of pulmonary circulation
	I30-I52	Other forms of heart disease
**Cerebrovascular disease**	I60-I69	Cerebrovascular diseases
**Vascular disease**	I70-I79	Diseases of arteries, arterioles and capillaries
	I80-I89	Diseases of veins, lymphatic vessels and lymph nodes, not elsewhere classified
	I95-I99	Other and unspecified disorders of the circulatory system

Each admission was aligned with a KRT treatment option of haemodialysis (HD), peritoneal dialysis (PD) or transplant based on diagnosis or procedure codes (Table S3). Relevant diagnosis and procedure codes were present for all patients for at least one admission a year but not all admissions contained a KRT relevant code.

Table S3: ICD 10AM diagnostic and procedure codes used to establish dialysis model of care

**ICD 10AM Procedure Code**
13100-00=Haemodialysis
13100-07=Intermittent PD long term
13100-08=Continuous PD long term
13109-01=Replacement of indwelling catheter
13110-00=Removal of indwelling catheter
**ICD 10AM diagnosis Code**
Z49.1=Extracorporial dialysis
Z94.0=Kidney transplant status
**AR-DRG Code**
L61Z=HD
L68Z=PD

To determine whether an individual relocated for treatment and whether any treatment was received at or closer to home, we identified home address (to suburb level) for each admission episode that occurred in the 24 months prior to commencement of KRT, taking the earliest admission address as the residence pre-KRT start. Less than 1% of patients did not have an admission in the 24 months prior to KRT start and their home address was taken as the address when they started KRT.

Patients were categorized as 'Relocated' when they lived outside the urban areas of Darwin and Alice Springs prior to commencement of KRT. All patients start KRT in the urban areas of Darwin and Alice Springs regardless of original residence. Limited capacity in rural and remote areas mean patients from these areas are not guaranteed treatment in or near their community and thus are placed on urban government housing priority lists once in the urban area. Hostel accommodation is usually available in the interim if not staying with family, although many patients never leave hostel accommodation due to the very long housing wait list. We defined 'Relocation' as having to change residence from rural/remote to urban, indefinitely, in order to access KRT.

Remoteness of a patient's home address was determined by mapping their residence (suburb or community) pre-KRT start to the Modified Monash Model (MMM), which classifies metropolitan, regional, rural and remote areas according to seven levels of geographical remoteness (3). That is: MMM1=Metropolitan city; MMM2=Outer regional centres; MMM3=Large rural towns; MMM4=Medium rural towns; MMM5=Small rural towns; MMM6=Remote community and MMM7=Very remote community. Only three

MMM categories apply to the NT: MMM2 Outer regional (which we renamed 'Urban' for the purposes of this study); MMM6: Remote and MMM7: Very remote.

We used the MMM for home residence as the Australian Government recently approved a Medicare Benefits Schedule (MBS) item (4), for staffed dialysis in very remote locations (MMM7). We were interested in understanding how many patients might possibly benefit from this item.

Activity was separated by region, Top End (TE) and Central Australia (CA), to align with health service responsibility in the NT.

Variable definitions (descriptions and calculations) are shown in Table S4.

Table S4: Description of available and created Outcome and Exposure variables in Final Study Population data set

Variable	Variable label	Value	Calculation	Type
year	Year of admission	Continuous (2008-2014)	Year of admission date	**Exposure**
indig	Ethnicity (Aboriginal)	Categorical (0= Not Aboriginal; 1=Aboriginal)	Hospital coding - collapsed to 'Aboriginal or Torres Strait Islander' or 'Not'	**Exposure**
gender	Gender (Male)	Categorical (0= Female; 1=Male)	Hospital coding	**Exposure**
region	Region (TE)	Categorical (0=Central Australia; 1=Top End)	Based on hospital coding	**Exposure**
Orig_district	Residence pre KRT stratified by NT Health Service district	Categorical (1=Darwin; 2= Darwin rural; 3=Tiwi Islands; 4=East Arnhem; 5=Daly West Arnhem; 6=Katherine; 7=Alice Urban; 8=Alice rural; 9=Barkly; 10=Central Desert; 11= Western Desert; 12=Interstate)	Based on hospital coding of Locality codes allocated to NT Health Service district	**Exposure**
Reloc_flag	Relocated	Categorical (0=Not relocated; 1=Relocated)	Flagged if NT Health Service District not equivalent to Darwin or Alice Springs	**Exposure**
Orig_MM7	Remoteness of residence pre-RRT start	Categorical (1=Outer regional; 2=Remote; 3=Very remote; 4=Interstate)	Modified Monash Model classification for areas of remoteness *Outer regional= Urban	**Exposure**
admage	Age at date of admission	Continuous (18-84)	Calculated from date of birth and date of admission	**Exposure**
admage_cat	Age at date of admission category	Categorical (0=<30yrs; 1=30-39yrs; 2=40-49yrs; 3=50-59yrs; 4=60-69yrs; 5=>70yrs)	Age at admission stratified into 6 categories	**Exposure**
RRT_start	Date of KRT commencement	Ordinal dd/mm/yyyy - Interval	Used as interval variable to calculate time on KRT and time in study	**Exposure**
dxtime_cat	Time on dialysis at time of admission category	Categorical (0=<1 yr; 1=>1-2yrs; 2=>2-3yrs; 3=>3-4yrs;	Calculated from KRT start date to date of admission and stratified by 12 month periods for first 5 years	**Exposure**
DxMoC	Dominant dialysis model of care	Categorical (0=DxMoC0; 1=DxMoC1; 2=DxMoC2; 3=DxMoC3; 4=DxMoC4; 5=DxMoC5; 6=DxMoC6)	Determined by majority model attendance over 3 week rolling period	**Exposure**
MoCyr_tar	Proportion of year exposed to DxMoC	Continuous (0-1.0)	Proportion of year spent in DxMoC (base unit =weeks) minus intermittent LTFU and time interstate	**Exposure**
MoCdxatt_cat	Category of calculated dialysis attendance by DxMoC/year	Categorical (1=High:>144; 2=Medium:132-143; 3=Low:<132)	Dialysis attendance stratified into high, medium and low attendance	**Exposure**

Table S4: Description of available and created Outcome and Exposure variables (continued)

Variable	Variable label	Value	Calculation	Type
DM	Diabetes Mellitus	Categorical (0=No; 1=Yes)	Presence of ICD-10AM code; if present always present	**Exposure**
CVD	Cerebrovascular Disease	Categorical (0=No; 1=Yes)	Presence of ICD-10AM code; if present always present	**Exposure**
CAD	Cardiovascular Disease	Categorical (0=No; 1=Yes)	Presence of ICD-10AM code; if present always present	**Exposure**
Hyptn	Hypertension	Categorical (0=No; 1=Yes)	Presence of ICD-10AM code; if present always present	**Exposure**
Obesity	Obesity	Categorical (0=No; 1=Yes)	Presence of ICD-10AM code; if present always present	**Exposure**
VascD	Vascular Disease	Categorical (0=No; 1=Yes)	Presence of ICD-10AM code; if present always present	**Exposure**
iLTFU	Intermittent lost to follow up	Categorical (0=No; 1=Yes)	Flagged for each week if attendance data (dialysis and hospital) is absent => 52 weeks for haemodialysis patients	**Outcome**
ltfu_time	Period of intermittent LTFU time	Continuous (0-2.75)	Calculated from intermittent LTFU, minimum of 52 weeks	**Used in calculation of time at risk**
calcMoC_att	Calculated outpatient dialysis attendance by DxMoC/year	Continuous (0-156)	Number of dialysis treatments by DxMoC, divided by time (weeks) in DxMoC x 52 (weeks) to represent yearly rate	**Outcome**
MoChosp_adm	Rate of hospital admissions by DxMoC/year	Continuous (0-40)	Sum of overnight admissions while exposed to a DxMoC	**Outcome**
yrlyhosp_adm	Annual rate of hospital admissions	Continuous (0-40)	Sum of overnight admissions by year per patient	**Outcome**
MoCEDpres	Rate of ED presentations by DxMoC/year	Continuous (0-50)	Sum of Emergency Department presentations while exposed to a DxMoC	**Outcome**
yrlyED_pres	Annual rate of ED presentations	Continuous (0-50)	Sum of Emergency Department presentations by year per patient	**Outcome**
misdx_mocyr	Admissions associated with missed dialysis by DxMoC/year	Continuous (0-50)	Sum of ICD codes for fluid overload or hyperkalaemia while exposed to a model of care	**Outcome**
misdx_cat	Risk for missed dialysis category	Categorical 0<5; 1 ≥5 High risk for missed dialysis	Based on 5 or more admissions with ICD codes E87.7 or E87.5 present per DxMoC exposure per year	**Outcome**
MoC_los	Number of inpatient days by DxMoC/year	Continuous (0-365)	Total days as inpatient while exposed to a DxMoC	**Outcome**
yrly_los	Number of inpatient days /year	Continuous (0-365)	Total days as inpatient by year per patient	**Outcome**
LTFU	Lost to follow-up	Categorical (0=No; 1=Yes)	Based on 'missing' data to end of observation period with no indication of death	**Censoring**
stdy_status	Status at end of study	Categorical (1=Alive; 2=Dead; 3=LTFU)	Censored at end of study as Alive, Dead or LTFU	**Outcome**

Results

Table S5 identifies the proportion of Aboriginal patients with a select comorbid condition at admission by DxMoC between the years 2008-2014. Patients can have more than one comorbid condition.

Table S5: Proportion of admissions (95% CI) for NT Aboriginal patient with a specific comorbid condition by DxMoC, 2008-2014

DxMoC % (95% CI)	Diabetes	Cardiac	Vascular	Obesity	Hypertension	Cerebrovascular
Incentre DxMoC0	80 (77-82)	57 (54-60)	45 (42-48)	13 (11-16)	95 (94-97)	7 (5-8)
Urban DxMoC1	86 (85-87)	61 (59-63)	59 (57-60)	18 (17-20)	98 (97-98)	8 (7-9)
Rural DxMoC2	89 (86-91)	69 (65-72)	58 (54-62)	12 (10-15)	97 (96-98)	3 (2-4)
Remote DxMoC3	83 (76-89)	61 (52-69)	55 (47-64)	10 (6-17)	100 (97-100)	4 (2-9)
RemoteCC* DxMoC4	92 (88-95)	55 (54-61)	61 (55-66)	19 (14-26)	98 (96-99)	8 (5-11)
SC HD* DxMoC5	87 (82-91)	76 (70-81)	61 (55-68)	16 (12-21)	94 (91-97)	7 (4-10)
SC PD* DxMoC6	74 (69-79)	60 (54-65)	60 (54-65)	11 (8-15)	97 (94-98)	6 (4-10)

**CC:Community-controlled; SC HD: self-care haemodialysis; SC PD: self-care peritoneal dialysis*

Dialysis Attendance

Table S6 displays the contribution of individual exposure variables to the mean annual dialysis attendance for patients receiving haemodialysis, therefore DxMoC6 (selfcare peritoneal dialysis) is not included.

Table S6: Mean annual dialysis treatments by exposure variables for NT Aboriginal patients 2008-2014

		Annual dialysis treatments	
**Univariate linear regression**			
		Mean (95% CI)	P value
**Gender**	Female	132 (131-133)	Ref
	Male	130 (128-131)	0.007
**Region**	Central Australia	127 (126-129)	Ref
	Top End	136 (135-138)	<0.001
**Residence pre-KRT**	Urban	135 (129-141)	Ref
	Remote	130 (126-133)	0.044
	Very Remote	131 (130-133)	0.235
	Interstate	115 (100-130)	0.008
**Admission age**	<30 years	124 (118-130)	Ref
	30-39	125 (121-128)	0.904
	40-49	129 (127-131)	0.162
	50-59	131 (129-133)	0.036
	60-69	137 (135-139)	<0.001
	>70	141 (136-146)	<0.001
**Time on dialysis**	<12mths	124 (121-128)	Ref
	1-2 yrs	125 (122-128)	0.747
	2-3yrs	129 (126-132)	0.045
	3-4yrs	130 (127-132)	0.025
	4-5yrs	134 (130-137)	<0.001
	>5yrs	136 (134-137)	<0.001
**DxMoC**	Incentre DxMoC0	97 (92-101)	Ref
	Urban DxMoC1	131 (129-132)	<0.001
	Rural DxMoC2	143 (140-145)	<0.001
	Remote DxMoC3	142 (136-147)	<0.001
	RemoteCC* DxMoC4	147 (141-152)	<0.001
	SC HD* DxMoC5	129 (124-133)	<0.001

**CC:Community-controlled; SC HD: self-care haemodialysis*

Dialysis attendance, hospital admissions and ED presentations

Low dialysis attendance is associated with increased rates of hospitalisations and ED presentations as shown in Table S7.

Table S7: Hospital admission and ED presentation incidence rate ratios for NT Aboriginal haemodialysis patients including dialysis attendance as an exposure variable, 2008-2014

Negative binomial regression adjusted for included variables		Multivariate Incidence Rate Ratio (IRR): Hospital admissions		Multivariate Incidence Rate Ratio (IRR): ED Presentations	
		IRR (95% CI)	P Value	IRR (95% CI)	P Value
Gender	Male (vs Females)	0.99 (0.93-1.06)	0.664	1.09 (0.97-1.22)	0.133
Region	TE (vs CA)*	0.80 (0.74-0.86)	<0.001	0.37 (0.32-0.43)	<0.001
Residence pre KRT	Urban	1	Reference	1	Reference
	Remote	1.38 (1.18-1.60)	<0.001	2.21 (1.57-3.11)	<0.001
	Very Remote	1.30 (1.16-1.50)	<0.001	2.01 (1.47-2.71)	<0.001
	Interstate	1.81 (1.34-2.83)	0.002	3.35 (1.76-6.35)	<0.001
Admission age	<30yrs	1	Reference	1	Reference
	30-39yrs	1.10 (0.90-1.33)	0.282	1.36 (0.96-1.94)	0.083
	40-49yrs	0.75 (0.62-0.90)	0.002	0.71 (0.51-0.99)	0.050
	50-59yrs	0.70 (0.58-0.85)	<0.001	0.58 (0.41-0.82)	0.002
	60-69yrs	0.78 (0.64-0.95)	0.010	0.70 (0.49-0.99)	0.048
	>70yrs	0.74 (0.57-0.91)	0.013	0.49 (0.31-0.77)	0.002
Time on dialysis	<12mths	1	Reference	1	Reference
	1-2 yrs	0.70 (0.65-081)	<0.001	1.10 (0.90-1.36)	0.355
	2-3yrs	0.65 (0.61-0.78)	<0.001	1.15 (0.92-1.44)	0.209
	3-4yrs	0.68 (0.60-0.77)	<0.001	1.13 (0.89-1.43)	0.301
	4-5yrs	0.59 (0.51-0.67)	<0.001	1.08 (0.84-1.39)	0.547
	>5yrs	0.65 (0.58-0.76)	<0.001	1.05 (0.86-1.30)	0.587
Dialysis attendance	High >=144	1	Reference	1	Reference
	Medium 132-143	1.37 (1.25-1.51)	<0.001	1.88 (1.58-2.58)	<0.001
	Low <=131	2.10 (1.96-2.28)	<0.001	3.29 (2.86-3.80)	<0.001
Comorbid conditions	Diabetes	1.27 (1.15-1.40)	<0.001	1.06 (0.88-1.27)	0.521
	Cardiac	1.34 (1.25-1.44)	<0.001	1.52 (1.34- 1.73)	<0.001
	Vascular	1.35 (1.26-1.45)	<0.001	1.06 (0.93-1.21	0.366
	Obesity	1.09 (1.00-1.18)	0.038	1.18 (1.02-1.36)	0.027
DxMoC	Incentre DxMoC0	1.91 (1.74-2.12)	<0.001	1.19 (0.99-1.43)	0.060
	Urban DxMoC1	1	Reference	1	Reference
	Rural DxMoC2	1.11 (1.00-1.21)	0.040	0.45 (0.37-0.56)	<0.001
	Remote DxMoC3	0.69 (0.55-0.86)	0.001	0.17 (0.07-0.39)	<0.001
	RemoteCC* DxMoC4	0.71 (0.60-085)	<0.001	0.69 (0.52-0.92)	0.013
	SC HD* DxMoC5	0.56 (0.47-0.66)	<0.001	0.34 (0.24-0.50)	<0.001

**TE: Top End; CA: Central Australia; *CC:Community-controlled; SC HD: self-care haemodialysis*

ED Presentations

When examining ED presentations, the unadjusted analysis showed increased rates associated with male gender (IRR=1.18, 95% CI:1.05-1.32) compared to female, remoteness of residence pre-KRT start, time on dialysis greater than 12 months, and the comorbidities of diabetes, vascular disease, cardiac disease and obesity (Table S8). However, on multivariate analysis, time on dialysis was not significant and the only comorbidity that remained significant was cardiac disease with an IRR=1.44; 95% CI: 1.26-1.64. Remoteness of residence pre-KRT start was associated with increased rates of ED presentations, while the rates were substantially lower for rural, remote and self-care models (DxMoC2-6) (Table S8).

This suggests that relocated people receiving care in the urban area had higher rates of ED presentations compared to people who were able to return to their communities and receive care at or closer to home in DxMoC2-6. However, we acknowledge that people in remote areas have little chance of presenting to a local emergency department, and if requiring care will be medically transferred to the urban hospital. Our data set did not include information about emergency medical transfers. However, if a medical transfer was warranted, it would result in an admission to hospital and as shown in Table 5 in the manuscript, the rates of hospital admissions for remote models was lower than urban models. We do not believe there has been a significant underestimation of ED use by patients attending rural and remote models.

Table S8: ED presentation incident rate ratios (IRR) for NT Aboriginal patients, 2008-2014

Negative binomial regression adjusted for included variables		ED Presentations Incidence Rate Ratio (IRR) Univariate		Multivariate	
		IRR (95% CI)	P value	IRR (95% CI)	P Value
Gender	Male (vs Females)	1.18 (1.05-1.32)	0.003	1.17 (1.04-1.32)	0.009
Region	TE (vs CA)*	0.25 (0.22-0.28)	<0.001	0.32 (0.28-0.37)	<0.001
Residence pre KRT	Urban	1	Reference	1	Reference
	Remote	6.67 (5.07-8.79)	<0.001	2.56 (1.81-3.61)	<0.001
	Very Remote	4.13 (3.25-5.26)	<0.001	2.40 (1.77- 3.26)	<0.001
	Interstate	15.32 (7.87-29.85)	<0.001	4.15 (2.13-8.09)	<0.001
Admission age	<30yrs	1	Reference	1	Reference
	30-39yrs	1.97 (1.44-2.70)	<0.001	1.41 (0.99-2.00)	0.052
	40-49yrs	0.80 (0.60-1.08)	0.144	0.66 (0.47-0.91)	0.020
	50-59yrs	0.64 (0.48-0.85)	0.002	0.53 (0.38-0.75)	<0.001
	60-69yrs	0.70 (0.52-0.95)	0.022	0.58 (0.41-0.82)	0.003
	>70yrs	0.39 (0.26-0.59)	<0.001	0.30 (0.19-0.47)	<0.001
Time on dialysis	<12mths	1	Reference		Reference
	1-2 yrs	2.04 (1.69-2.46)	<0.001	1.12 (0.91-1.39)	0.278
	2-3yrs	1.9 (1.56-2.34)	<0.001	1.09 (0.87-1.37)	0.428
	3-4yrs	1.79 (1.45-2.21)	<0.001	1.06 (0.83-1.34)	0.628
	4-5yrs	1.86 (1.48-2.32)	<0.001	0.97 (0.75-1.26)	0.85
	>5yrs	1.60 (1.37-1.87)	<0.001	0.85 (0.69-1.05)	0.135
Comorbid conditions	Diabetes	0.967 (0.83-1.12)	<0.001	0.99 (0.83-1.19)	0.972
	Cardiac	1.35 (1.20-1.51)	<0.001	1.44 (1.26-1.64)	<0.001
	Vascular	1.37 (1.22-1.53)	<0.001	1.13 (0.99-1.29)	0.064
	Obesity	1.87 (1.62-2.15)	<0.001	1.13 (0.98-1.32)	0.104
DxMoC	Incentre DxMoC0	1.19 (0.98-1.44)	0.070	1.42 (1.17-1.71)	<0.001
	Urban DxMoC1	1	Reference	1	Reference
	Rural DxMoC2	0.30 (0.24-0.37)	<0.001	0.34 (0.27-0.41)	<0.001
	Remote DxMoC3	0.04 (0.02-0.09)	<0.001	0.11 (0.05-0.26)	<0.001
	RemoteCC* DxMoC4	0.72 (0.53-0.99)	0.041	0.57 (0.42-0.77)	<0.001
	SC HD* DxMoC5	0.19 (0.13-0.28)	<0.001	0.33 (0.23-0.48)	<0.001
	SC PD* DxMoC6	0.21 (0.16-0.29)	<0.001	0.29 (0.21-0.40)	<0.001

**TE: Top End; CA: Central Australia; CC: Community-controlled; SC HD: self-care haemodialysis; SC PD: self-care peritoneal dialysis*

Days in hospital

We also examined the total annual days in hospital (Table S9). Most exposure variables tested individually were statistically significant. The mean annual days were higher for those with a comorbidity of diabetes, cardiac disease, vascular disease and obesity compared to those without the respective comorbidity, while dialysing in remote and self-care HD models (DxMoC3 to 5) was associated with lower mean annual days in hospital compared to Incentre DxMoC0. When modelled together the associations persisted although the difference in days in hospital for those with and without obesity were fewer. Self-care PD DxMoC6 had relatively high adjusted mean annual days in hospital at 24.1 (95% CI:22.9-25.4) compared to Incentre DxMoC0 of 18.3 (95% CI:17.5-19.0) days in hospital (Table S9).

An analysis of diagnosis codes for this group noted a higher rate of admissions for more severe and complex conditions when compared to the other models of care (5).

Table S9: Mean annual days in hospital for NT Aboriginal patients 2008-2014

**Zero inflated poisson regression adjusted for included variables**		**Days in hospital** Univariate		Multivariate	
		Mean (95% CI)	P value	Mean (95% CI)	P Value
Gender	Female	17.7 (17.2-18.1)	Reference	17.6 (17.2-17.9)	Reference
	Male	16.2 (15.7-16.6)	<0.001	16.3 (15.9-16.7)	<0.001
Region	Central Australia	18.2 (17.8-18.6)	Reference	18.2 (17.7-18.5)	Reference
	Top End	15.9 (15.5-16.3)	<0.001	16.0 (15.6-16.4)	<0.001
Residence pre-KRT	Urban	13.9 (13.0-14.7)	Reference	15.5 (13.8-17.3)	Reference
	Remote	17.1 (16.4-17.9)	<0.001	15.3 (14.4-16.3)	<0.001
	Very Remote	17.7 (17.3-18.0)	<0.001	17.7 (17.2-18.1)	<0.001
	Interstate	16.1 (12.2-20.0)	0.567	18.6 (14.2-22.9)	0.197
Admission age	<30 years	13.6 (12.2-14.9)	Reference	17.5 (16.2-18.9)	Reference
	30-39	16.6 (15.8-17.5)	<0.001	18.1 (17.3-18.9)	0.582
	40-49	14.9 (14.4-15.4)	0.005	15.6 (15.1-16.1)	0.001
	50-59	17.3 (16.7-17.8)	<0.001	16.6 (16.1-17.1)	0.164
	60-69	20.1 (19.3-21.0)	<0.001	18.5 (17.8-19.2)	<0.001
	>70	17.9 (16.4-19.4)	<0.001	18.2 (16.8-19.5)	0.004
Time on dialysis	<12mths	13.8 (13.3-14.3	Reference	16.2 (15.9-17.1)	Reference
	1-2 yrs	16.7 (16.0-17.5)	<0.001	18.3 (17.6-19.1)	<0.001
	2-3yrs	15.8 (14.9-16.7)	<0.001	16.4 (15.6-17.2)	<0.001
	3-4yrs	18.5 (17.4-19.7)	<0.001	18.8 (17.8-19.8)	<0.001
	4-5yrs	15.4 (14.3-16.4)	<0.001	14.5 (13.6-15.5)	<0.001
	>5yrs	19.4 (18.8-20.0)	<0.001	16.9 (16.4-17.4)	<0.001
Comorbid conditions	Diabetes (No)	11.2 (10.6-11.7)	Reference	12.0 (11.4-12.6)	Reference
	Yes	18.4 (18.0-18.6)	<0.001	18.1 (17.8-18.5)	<0.001
	Cardiac (No)	12.7 (12.3-13.1)	Reference	14.3 (13.8-14.7)	Reference
	Yes	19.8 (19.4-20.2)	<0.001	18.5 (18.1-18.9)	<0.001
	Vascular (No)	10.6 (10.3-11.0)	Reference	11.2 (10.8-11.6)	Reference
	Yes	22.1 (21.6-22.5)	<0.001	21.2 (20.8-21.7)	<0.001
	Obesity (No)	16.3 (16.0-16.6)	Reference	16.8 (16.5-17.1)	Reference
	Yes	21.0 (20.1-21.9)	<0.001	18.2 (17.4-19.0)	<0.001
DxMoC	Incentre DxMoC0	16 (15.3-16.6)	Reference	18.3 (17.5-19.0)	Reference
	Urban DxMoC1	18.3 (17.9-18.8)	<0.001	17.6 (17.2-18.1)	<0.007
	Rural DxMoC2	17.3 (16.4-18.3)	<0.001	16.0 (15.1-16.9)	<0.001
	Remote DxMoC3	13.6 (11.5-15.7)	<0.001	14.6 (12.8-16.5)	<0.001
	RemoteCC* DxMoC4	5.9 (5.0-6.8)	<0.001	5.2 (4.3 -6.0)	<0.001
	SC HD* DxMoC5	11.1 (9.9-12.4)	<0.001	12.1 (10.9-13.3)	<0.001
	SC PD* DxMoC6	22.5 (21.1-23.8)	<0.001	24.1 (22.9-25.4)	<0.001

**CC:Community-controlled; SC HD: self-care haemodialysis; SC PD: self-care peritoneal dialysis*

References

1. Australian Statistical Geography Standard (ASGS) [Internet]. ABS. 2016 [cited 22 August 2018]. Available from: Australian Statistical Geography Standard (ASGS) Volume 5 – Remoteness Structure (cat. no. 1270.0.55.005) publication.

2. Australian Institute of Health and Welfare. Australian hospital peer groups. Health services series no 66 Cat no HSE 170. Canberra: AIHW; 2015.

3. Department of Health. Modified Monash Model: Australian Government; 2015 [updated 12 June 2018,. Available from: http://www.health.gov.au/internet/main/publishing.nsf/content/modified-monash-model.

4. Australian Government. Medicare Benefits Schedule: Department of Health; 2018 [updated 13 December 2018. Available from: http://www.mbsonline.gov.au/internet/mbsonline/publishing.nsf/Content/Home.

5. Gorham G, Howard K, Cunningham J, Barzi F, Lawton P, Cass A. Do remote dialysis services really cost more? An economic analysis of hospital and dialysis modality costs associated with dialysis services in urban, rural and remote settings. BMC Health Serv Res. 2021;21(1):582.
